# Ilaprazole Versus Esomeprazole for Artificial Ulcer Healing After Gastric Endoscopic Submucosal Dissection: A Single-Center Retrospective Study

**DOI:** 10.3390/jcm15093357

**Published:** 2026-04-28

**Authors:** Dae-Gon Ryu, Su-Jin Kim, Su-Bum Park, Jin-Ook Jang, Woo-Jin Kim, Cheol-Min Lee, Jin-Hyuck Cho, Eun-Jung Choi, Cheol-Woong Choi

**Affiliations:** Department of Internal Medicine, Pusan National University School of Medicine, Research Institute for Convergence of Biomedical Science and Technology, Pusan National University Yangsan Hospital, Yangsan 50612, Republic of Korea; gon32gon@pusan.ac.kr (D.-G.R.); endoksj@pusan.ac.kr (S.-J.K.); psubumi@pusan.ac.kr (S.-B.P.); a_warm_mind@naver.com (J.-O.J.); pangolinlego@naver.com (W.-J.K.); oyatrhee@gmail.com (C.-M.L.); med92447@gmail.com (J.-H.C.); drchoi91@pusan.ac.kr (E.-J.C.)

**Keywords:** endoscopic submucosal dissection, artificial ulcer healing, ilaprazole, proton pump inhibitors, delayed bleeding, gastric neoplasia

## Abstract

**Background**: Endoscopic submucosal dissection (ESD) is widely used for the treatment of gastric neoplasia; however, the large artificial ulcer created during the procedure requires several weeks to heal. Although proton pump inhibitors (PPIs) are routinely administered after ESD, evidence comparing individual PPIs for artificial ulcer healing in real-world practice remains limited. This study compared the effectiveness of oral ilaprazole and oral esomeprazole as maintenance therapy after gastric ESD. **Methods**: This retrospective single-center study included patients who underwent gastric ESD between January 2020 and December 2024. All patients received intravenous PPI therapy for two days after ESD and were subsequently prescribed either oral ilaprazole 20 mg once daily or esomeprazole 40 mg once daily for 8 weeks. The primary outcome was complete artificial ulcer healing at 8 weeks. The secondary outcome was post-discharge delayed bleeding. **Results**: A total of 229 patients were analyzed (147 in the esomeprazole group and 82 in the ilaprazole group). The overall 8-week ulcer healing rate was 94.3%, with no significant difference between the ilaprazole and esomeprazole groups (97.5% vs. 92.5%, *p* = 0.114). In multivariate analysis, artificial ulcer size ≥ 30 mm was the only independent predictor of incomplete ulcer healing (odds ratio 20.850, 95% confidence interval 1.884–230.712, *p* = 0.013). Post-discharge delayed bleeding occurred in 8 patients (3.4%), all in the esomeprazole group (*p* = 0.032). No treatment-related adverse events were observed. **Conclusions**: Ilaprazole demonstrated ulcer-healing efficacy comparable to esomeprazole after gastric ESD. Artificial ulcer size ≥ 30 mm was the principal determinant of delayed healing, whereas the treatment group was not independently associated with healing outcomes. Ilaprazole may be considered a reasonable maintenance PPI option in routine post-ESD management.

## 1. Introduction

Endoscopic submucosal dissection (ESD) has become an established and widely accepted therapeutic procedure for the management of gastric epithelial neoplasia, enabling en bloc resection and high curative rates even for large or ulcerative lesions [1,2]. However, unlike endoscopic mucosal resection, ESD inevitably creates a large and deep artificial ulcer involving the submucosal layer, which requires a prolonged healing process and careful post-procedural management [2,3]. Artificial ulcers induced by ESD differ fundamentally from conventional peptic ulcers in their pathophysiology. Histopathologic studies have demonstrated that ESD-induced ulcers are characterized by submucosal dissection with relative preservation of the proper muscle layer, followed by early ulcer contraction and subsequent epithelial regeneration [4,5]. Most artificial ulcers heal within 8 weeks under standard acid suppression therapy, regardless of their initial location, although delayed healing has been reported in cases with large resection size or severe submucosal fibrosis [5,6,7]. Among ESD-related adverse events, post-discharge delayed bleeding remains a clinically important complication because it may result in hematemesis, emergency endoscopy, transfusion, or prolonged hospitalization [6]. Previous studies have reported delayed bleeding rates ranging from approximately 3% to 11%, with bleeding risk largely influenced by lesion size, procedural complexity, and the use of antithrombotic agents rather than by the choice of acid-suppressive therapy alone [6,8,9].

Acid suppression therapy is routinely administered after gastric ESD to facilitate artificial ulcer healing and reduce procedure-related complications [7,10]. Proton pump inhibitors (PPIs) are currently regarded as the standard of care; however, the optimal selection of PPIs in the post-ESD setting remains unclear, as most treatment strategies have been extrapolated from data on conventional gastric ulcers rather than derived from ESD-specific evidence [10,11]. Ilaprazole is a long-acting PPI with minimal dependence on CYP2C19 metabolism and a relatively prolonged plasma half-life, resulting in stable and sustained acid suppression [12,13]. These pharmacokinetic properties suggest that ilaprazole may be an effective option for post-ESD acid suppression. However, clinical data comparing ilaprazole with other PPIs for artificial ulcer healing after gastric ESD in real-world practice remain limited. Therefore, this study aimed to compare ilaprazole and esomeprazole with respect to 8-week artificial ulcer healing after gastric ESD and to identify clinical and endoscopic factors associated with delayed ulcer healing and post-discharge delayed bleeding.

## 2. Materials and Methods

### 2.1. Study Design and Patients

This single-center retrospective study was conducted at a tertiary referral hospital and included patients who underwent gastric endoscopic submucosal dissection (ESD) between January 2020 and December 2024. During the study period, a total of 458 gastric ESD procedures were performed for gastric adenoma, early gastric cancer, or subepithelial tumors, which represent standard indications for gastric ESD.

**Figure 1:**
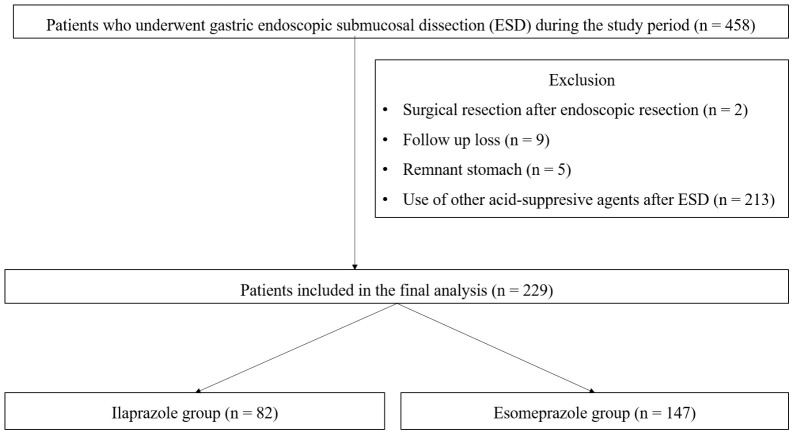
Flow diagram of patient selection based on acid-suppressive therapy prescribed at discharge after gastric ESD. ESD, endoscopic submucosal dissection.

Patients were eligible for inclusion if they completed a planned 8-week follow-up endoscopy after ESD. The 8-week time point was selected based on previous studies demonstrating that most artificial ulcers induced by ESD reach complete healing or the scar stage within this period under PPI therapy [4,10]. At discharge after ESD, all patients were prescribed a single oral acid-suppressive agent. Patients were excluded if they underwent subsequent surgical resection after ESD (*n* = 2), were lost to follow-up before the scheduled 8-week endoscopy (*n* = 9), had remnant stomach status (*n* = 5), or were prescribed PPIs or potassium-competitive acid blockers other than ilaprazole or esomeprazole at discharge (*n* = 213). Treatment groups were defined based on the oral PPI regimen initiated after completion of a standardized intravenous esomeprazole induction phase during the first two postoperative days. After applying these criteria, 229 ESD procedures were included in the final analysis, comprising 82 patients in the ilaprazole group and 147 patients in the esomeprazole group. A detailed flow diagram of patient inclusion and exclusion is shown in Figure 1.

All ESD procedures were performed by a single experienced endoscopist (Cheol Woong Choi), ensuring procedural consistency and minimizing operator-related variability. This study was conducted in accordance with the Declaration of Helsinki and was approved by the Institutional Review Board of Pusan National University Hospital (IRB No. 55-2025-037). The requirement for informed consent was waived owing to the retrospective nature of the study.

### 2.2. ESD Procedures

ESD was performed using a high-definition gastroscope (GIF-H290) or a multibending endoscope (GIF-2TQ260M; Olympus, Tokyo, Japan), selected according to lesion location and endoscopic accessibility. Standard ESD knives, including H-, O-, and I-type ClearCut knives (Finemedix, Daegu, Republic of Korea), were used. The I-type knife was selectively applied in cases with marked submucosal fibrosis, which has been reported to increase procedural difficulty and prolong dissection time.

The ESD procedure followed a standardized sequence: circumferential marking around the lesion using spray coagulation mode, mucosal incision along the marking line, and submucosal dissection with repeated submucosal injections as needed. During submucosal dissection, visible vessels were prophylactically coagulated to reduce the risk of delayed bleeding, in accordance with established ESD practice [8]. A routine second-look endoscopy was performed on postoperative day 1, and additional endoscopic hemostasis was applied when exposed vessels, adherent clots, or active oozing were identified, although the preventive effect of second-look endoscopy remains controversial [6].

### 2.3. Periprocedural Management and Acid Suppression Therapy

All patients received intravenous esomeprazole 40 mg twice daily from the day of ESD until postoperative day 2. In the absence of immediate post-procedural bleeding or other complications, oral acid suppression therapy was initiated on postoperative day 2 with either ilaprazole 20 mg once daily or esomeprazole 40 mg once daily and continued for 8 weeks. The choice between ilaprazole and esomeprazole was determined at the discretion of the endoscopist, as no institutional guideline preferentially recommended one PPI over the other during the study period. Ilaprazole is a long-acting PPI with minimal dependence on CYP2C19 metabolism and a prolonged plasma half-life, resulting in relatively stable acid suppression compared with some conventional PPIs [12,13].

### 2.4. Lesion Characteristics and Outcome Definitions

Lesion characteristics were assessed using diagnostic and intraoperative endoscopy and included ulceration, erosion, submucosal fibrosis, central depression, nodular surface, and mucosal redness. Submucosal fibrosis was defined as clearly visible fibrotic strands during dissection or insufficient mucosal elevation after submucosal injection. Procedure time was measured from the initiation of circumferential marking to completion of final hemostasis. Artificial ulcer size was determined by measuring the maximal diameter of the resected specimen, which was pinned flat on a board immediately after ESD and measured using a standard ruler.

At the scheduled follow-up endoscopy performed approximately 8 weeks after ESD (7–9 weeks), complete ulcer healing was defined as full re-epithelialization without a residual ulcer base, whereas incomplete healing was defined as the persistence of a mucosal defect. Bleeding events were classified as in-hospital bleeding (occurring within postoperative days 0–2) or post-discharge delayed bleeding (occurring from postoperative day 3 onward). Bleeding was diagnosed when clinical evidence of hematemesis or melena was present. All bleeding events were confirmed and managed endoscopically when required. Post-discharge delayed bleeding was defined only in patients who presented to the emergency department with hematemesis or melena and underwent emergency endoscopic evaluation.

### 2.5. Outcome Measures

The primary outcome was complete healing of artificial ulcers at 8 weeks after gastric ESD. The secondary outcome was the occurrence of post-discharge delayed bleeding. Adverse events related to acid-suppressive therapy were also recorded.

### 2.6. Statistical Analysis

Categorical variables were compared using the chi-square test or Fisher’s exact test, as appropriate, and continuous variables were compared using Student’s *t*-test. Univariate logistic regression analysis was performed to identify factors associated with incomplete ulcer healing. Variables with a *p*-value < 0.05 in univariate analysis were entered into multivariate logistic regression analysis. Odds ratios (ORs) and 95% confidence intervals (CIs) were calculated. A two-sided *p*-value < 0.05 was considered statistically significant. All statistical analyses were performed using SPSS version 27.0 (IBM Corp., Armonk, NY, USA).

## 3. Results

### 3.1. Baseline Characteristics (Table 1)

A total of 229 gastric ESD procedures were included in the final analysis, comprising 82 patients in the ilaprazole group and 147 patients in the esomeprazole group. The patient selection and exclusion process is summarized in Figure 1. Baseline demographic and clinical characteristics are presented in Table 1. Patients in the ilaprazole group were younger than those in the esomeprazole group (65.1 ± 9.6 vs. 68.2 ± 9.0 years, *p* = 0.018). The proportion of male patients did not differ significantly between the two groups (68.3% vs. 65.3%, *p* = 0.646). With respect to comorbidities, hypertension was more prevalent in the ilaprazole group than in the esomeprazole group (24.4% vs. 13.7%, *p* = 0.042), whereas the prevalence of diabetes mellitus was comparable between the two groups (51.2% vs. 52.7%, *p* = 0.825). The use of antithrombotic medications, including antiplatelet agents, anticoagulants, and dual antiplatelet or anticoagulant therapy, did not differ significantly between the two groups.

**Table 1:** Baseline characteristics of patients after gastric ESD.

Variable	Esomeprazole (*n* = 147)	Ilaprazole (*n* = 82)	*p* Value
Demographics			
Age, years (mean ± SD)	68.2 ± 9.0	65.1 ± 9.6	0.018
Male sex, *n* (%)	96 (65.3)	56 (68.3)	0.646
Medications			
Antiplatelet agent use, *n* (%)	28 (19.0)	20 (24.4)	0.341
Anticoagulant use, *n* (%)	7 (4.8)	2 (2.4)	0.386
Dual antiplatelet/anticoagulant therapy, *n* (%)	4 (2.7)	0 (0)	0.132
Comorbidities			
Hypertension, *n* (%)	20 (13.7)	20 (24.4)	0.042
Diabetes mellitus, *n* (%)	77 (52.7)	42 (51.2)	0.825

Values are presented as mean ± standard deviation (SD) or number (%). ESD, endoscopic submucosal dissection; SD, standard deviation.

### 3.2. Endoscopic Characteristics and Post-Procedural Clinical Outcomes (Table 2)

Endoscopic and procedural characteristics are summarized in Table 2. There were no significant differences between the ilaprazole and esomeprazole groups in terms of artificial ulcer size (27.9 ± 10.0 mm vs. 29.7 ± 11.7 mm, *p* = 0.233) or procedure time (9.6 ± 7.1 min vs. 11.8 ± 12.8 min, *p* = 0.151).

**Table 2:** Endoscopic characteristics and post-procedural clinical outcomes.

Variable	Esomeprazole (*n* = 147)	Ilaprazole (*n* = 82)	*p* Value
Lesion/procedure burden			
Artificial ulcer size, mm (mean ± SD)	29.7 ± 11.7	27.9 ± 10.0	0.233
Procedure time, min (mean ± SD)	11.8 ± 12.8	9.6 ± 7.1	0.151
Submucosal fibrosis, *n* (%)	16 (10.9)	2 (2.4)	0.023
Endoscopic morphology			
Ulceration, *n* (%)	2 (1.4)	2 (2.4)	0.550
Central depression, *n* (%)	44 (29.9)	23 (28.0)	0.764
Nodular surface, *n* (%)	31 (21.1)	13 (15.9)	0.335
Redness, *n* (%)	98 (66.7)	55 (67.1)	0.950
Erosion, *n* (%)	15 (10.2)	15 (18.3)	0.082
Post-ESD clinical outcomes			
Complete ulcer healing at 8 weeks, *n* (%)	136 (92.5)	80 (97.6)	0.114
In-hospital bleeding, *n* (%)	3 (2.0)	4 (4.9)	0.232
Post-discharge delayed bleeding, *n* (%)	8 (5.4)	0 (0)	0.032

Values are presented as mean ± standard deviation (SD) or number (%). ESD, endoscopic submucosal dissection; SD, standard deviation.

Submucosal fibrosis was observed less frequently in the ilaprazole group than in the esomeprazole group (2.4% vs. 10.9%, *p* = 0.023). Other endoscopic morphological features, including ulceration, central depression, nodular surface, and mucosal redness, did not differ significantly between the two groups. Erosion tended to be more frequent in the ilaprazole group; however, this difference did not reach statistical significance (18.3% vs. 10.2%, *p* = 0.082).

Regarding post-ESD clinical outcomes, complete artificial ulcer healing at follow-up was achieved in 97.6% of patients in the ilaprazole group and 92.5% of patients in the esomeprazole group, with no statistically significant difference between groups (*p* = 0.114). In-hospital bleeding occurred in 4 patients (4.9%) in the ilaprazole group and in 3 patients (2.0%) in the esomeprazole group (*p* = 0.232). Post-discharge delayed bleeding was observed in 8 patients (5.4%) in the esomeprazole group, whereas no delayed bleeding events occurred in the ilaprazole group (*p* = 0.032).

### 3.3. Univariate Analysis of Factors Associated with Incomplete Artificial Ulcer Healing (Table 3)

Univariate analysis of factors associated with incomplete artificial ulcer healing at follow-up is summarized in Table 3. Incomplete healing was observed in 13 patients (5.7%). Incomplete ulcer healing was not significantly associated with treatment group (ilaprazole vs. esomeprazole, *p* = 0.114) or sex (*p* = 0.704). In contrast, older age (≥65 years) was significantly associated with incomplete healing (92.3% vs. 61.6%, *p* = 0.026). Lesion- and procedure-related factors showed strong associations with healing outcomes. Artificial ulcer size ≥ 30 mm was significantly more frequent in patients with incomplete healing than in those with complete healing (92.3% vs. 45.4%, *p* = 0.001). Submucosal fibrosis was also more commonly observed in the incomplete healing group (23.1% vs. 6.9%, *p* = 0.036). A longer procedure time (≥10 min) showed a borderline association with incomplete healing but did not reach statistical significance (*p* = 0.054). Regarding endoscopic morphology, features such as central depression, nodular surface, redness, and erosion were not significantly associated with incomplete ulcer healing (all *p* > 0.05). Among post-procedural and clinical factors, post-discharge delayed bleeding was significantly associated with incomplete healing (15.4% vs. 2.8%, *p* = 0.016). Antiplatelet agent use was also more frequent in patients with incomplete healing (46.2% vs. 19.4%, *p* = 0.022). In addition, hypertension was significantly associated with incomplete healing (38.5% vs. 16.3%, *p* = 0.041). In contrast, Helicobacter pylori infection, anticoagulant use, diabetes mellitus, and prophylactic hemostasis during second-look endoscopy were not significantly associated with healing outcomes.

**Table 3:** Univariate analysis of factors associated with incomplete artificial ulcer healing at 8 weeks.

Variable	Incomplete Healing (*n* = 13)	Complete Healing (*n* = 216)	Total (*n* = 229)	*p* Value
Ilaprazole group, *n* (%)	2 (15.4)	80 (37.0)	82 (35.8)	0.114
Male sex, *n* (%)	8 (61.5)	144 (66.7)	152 (66.4)	0.704
Age ≥ 65 years, *n* (%)	12 (92.3)	133 (61.6)	145 (63.3)	0.026
Artificial ulcer size ≥ 30 mm, *n* (%)	12 (92.3)	98 (45.4)	110 (48.0)	0.001
Procedure time ≥ 10 min, *n* (%)	5 (38.5)	37 (17.1)	42 (18.3)	0.054
Submucosal fibrosis, *n* (%)	3 (23.1)	15 (6.9)	18 (7.9)	0.036
Central depression, *n* (%)	6 (46.2)	61 (28.2)	67 (29.3)	0.169
Nodular surface, *n* (%)	4 (30.8)	40 (18.7)	44 (19.2)	0.276
Redness, *n* (%)	10 (76.9)	143 (66.2)	153 (66.8)	0.425
Erosion, *n* (%)	1 (7.7)	29 (13.4)	30 (13.1)	0.552
Post-discharge delayed bleeding, *n* (%)	2 (15.4)	6 (2.8)	8 (3.5)	0.016
Prophylactic hemostasis during second-look endoscopy, *n* (%)	7 (53.8)	103 (47.7)	110 (48.0)	0.666
Helicobacter pylori infection, *n* (%)	6 (46.2)	95 (44.0)	101 (44.1)	0.878
Antiplatelet agent use, *n* (%)	6 (46.2)	42 (19.4)	48 (21.0)	0.022
Anticoagulant use, *n* (%)	0 (0)	9 (4.2)	9 (3.9)	0.453
Diabetes mellitus, *n* (%)	9 (69.2)	110 (51.2)	119 (52.2)	0.205
Hypertension, *n* (%)	5 (38.5)	35 (16.3)	40 (17.5)	0.041

Values are presented as a number (%). Incomplete ulcer healing was defined as a persistent mucosal defect at follow-up endoscopy performed approximately 8 weeks after ESD. ESD, endoscopic submucosal dissection.

### 3.4. Multivariate Analysis for Incomplete Artificial Ulcer Healing (Table 4)

**Figure 2:**
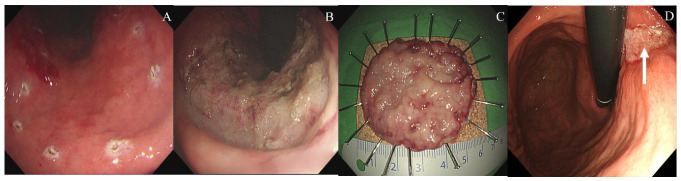
Illustrative case of incomplete artificial ulcer healing at 8-week follow-up in a patient treated with esomeprazole after gastric endoscopic submucosal dissection. (**A**) Initial endoscopic image showing a subtle erythematous and depressed lesion within the marking dots at the mid third of the stomach. (**B**) Artificial ulcer immediately after en bloc resection, demonstrating a large post-resection defect with visible submucosal exposure. (**C**) Resected specimen mounted and pinned for mapping, measuring approximately 60 mm in maximal diameter. Final histopathological examination confirmed tubular adenoma with high-grade dysplasia. (**D**) Follow-up endoscopy at 8 weeks shows incomplete ulcer healing, with a persistent whitish epithelial defect remaining at the resection site (white arrow).

**Figure 3:**
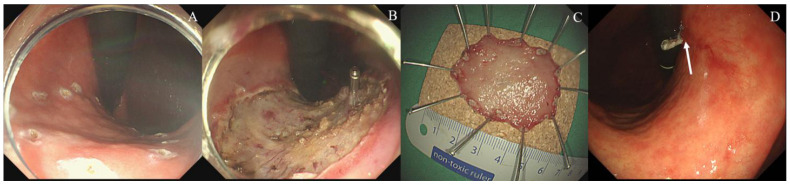
Illustrative case of complete artificial ulcer healing at 8-week follow-up in a patient treated with ilaprazole after gastric endoscopic submucosal dissection. (**A**) Pre-ESD endoscopic image showing a whitish, slightly elevated mucosal lesion within the marking dots. (**B**) Artificial ulcer immediately after ESD, demonstrating submucosal exposure. A clip was placed for suspected intraprocedural muscle-layer injury. (**C**) Resected specimen mounted and pinned for mapping, measuring approximately 50 mm in maximal diameter. Final histopathological examination confirmed low-grade dysplasia. (**D**) Follow-up endoscopy at 8 weeks showing complete ulcer healing, with a well-formed scar and the previously placed clip still visible (white arrow). ESD, endoscopic submucosal dissection.

In multivariate logistic regression analysis (Table 4), after adjustment for age ≥ 65 years, submucosal fibrosis, antiplatelet agent use, hypertension, and post-discharge delayed bleeding, artificial ulcer size ≥ 30 mm was the only independent predictor of incomplete artificial ulcer healing at 8 weeks (odds ratio [OR] 20.850, 95% confidence interval [CI] 1.884–230.712, *p* = 0.013). Representative endoscopic images of incomplete and complete artificial ulcer healing at 8-week follow-up are shown in Figure 2 and Figure 3.

**Table 4:** Multivariate analysis of factors associated with incomplete artificial ulcer healing at 8 weeks.

Variable	Odds Ratio (OR)	95% Confidence Interval	*p* Value
Age ≥ 65 years	3.815	0.450–32.322	0.219
Artificial ulcer size ≥ 30 mm	20.850	1.884–230.712	0.013
Submucosal fibrosis	2.078	0.395–10.943	0.388
Antiplatelet agent use	2.719	0.687–10.762	0.154
Hypertension	1.469	0.325–6.644	0.617
Post-discharge delayed bleeding	12.725	0.804–201.417	0.071

Multivariate logistic regression analysis was performed to identify independent predictors of incomplete artificial ulcer healing at approximately 8 weeks after gastric ESD. Variables included in the model were selected based on clinical relevance and univariate analysis results. OR, odds ratio; CI, confidence interval; ESD, endoscopic submucosal dissection.

### 3.5. Post-Discharge Delayed Bleeding (Table 5)

**Figure 4:**
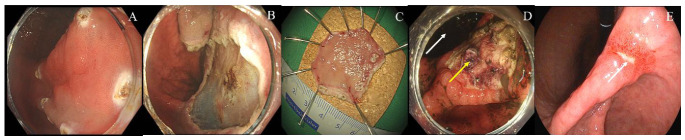
Illustrative case of post-discharge delayed bleeding and delayed artificial ulcer healing in a patient treated with esomeprazole after gastric endoscopic submucosal dissection. (**A**) Pre-ESD endoscopic image showing an erythematous and slightly depressed lesion within the marking dots on the lesser curvature of the antrum. (**B**) Artificial ulcer immediately after ESD, demonstrating a well-exposed submucosal resection base. (**C**) Resected specimen mounted and pinned for mapping, measuring approximately 30 mm in maximal diameter. Final histopathological examination revealed a well-differentiated tubular adenocarcinoma. (**D**) On post-ESD day 10, the patient presented with hematemesis. Emergency endoscopy demonstrated a large intragastric blood clot (white arrow) and a suspected bleeding focus with an adherent clot overlying the artificial ulcer (yellow arrow), and endoscopic coagulation hemostasis was successfully performed. (**E**) Follow-up endoscopy at 8 weeks showing delayed ulcer healing, with a persistent epithelial defect at the previous resection site (white arrow). ESD, endoscopic submucosal dissection.

Post-discharge delayed bleeding occurred in 8 patients (3.5%) and was observed exclusively in the esomeprazole group, whereas no bleeding events occurred in the ilaprazole group (*p* = 0.032). All bleeding events developed within 14 days after ESD and were successfully managed with endoscopic coagulation-based hemostasis. A representative case of post-discharge delayed bleeding after gastric ESD is shown in Figure 4.

**Table 5:** Univariate analysis of factors associated with post-discharge delayed bleeding after gastric ESD.

Characteristics	No Delayed Bleeding (*n* = 221)	Post-Discharge Delayed Bleeding (*n* = 8)	Total (*n* = 229)	
Ilaprazole group, *n* (%)	82 (37.1)	0 (0)	82 (35.8)	0.032
Complete ulcer healing at 8 weeks, *n* (%)	210 (95.0)	6 (75.0)	216 (94.3)	0.016
Male, *n*, %	144 (65.2)	8 (100)	152 (66.4)	0.040
Age, years, mean ± SD	66.8 (9.3)	74.6 (6.5)	67.1 (9.3)	0.021
Age ≥ 65 years, *n* (%)	137 (62.0)	8 (100)	145 (63.3)	0.028
Artificial ulcer size, mm (mean ± SD)	29.2 (11.3)	24.5 (7.3)	29.1 (11.2)	0.242
Procedure time, min, min ± SD	11.1 (11.3)	8.8 (3.4)	11.0 (11.2)	0.582
Procedure time ≥ 10 min	41 (18.6)	1 (12.5)	42 (18.3)	0.664
Endoscopic features, *n*, %				
Ulceration	4 (1.8)	0 (0)	4 (1.7)	0.701
Submucosal fibrosis	16 (7.2)	2 (25.0)	18 (7.9)	0.067
Central depression	64 (29.0)	3 (37.5)	67 (29.3)	0.602
Nodular surface	42 (19.0)	2 (25.0)	44 (19.2)	0.672
Redness	147 (66.5)	6 (75.0)	153 (66.8)	0.617
Erosion	28 (12.7)	2 (25.0)	30 (13.1)	0.310
In-hospital bleeding, *n* (%)	7 (3.2)	0 (0)	7 (3.1)	0.609
Prophylactic hemostasis during 2nd-look endoscopy, *n* (%)	105 (47.5)	5 (62.5)	110 (48.0)	0.405
H. pylori infection, *n* (%)	98 (44.3)	3 (37.5)	101 (44.1)	0.702
Final histology, *n* (%)				0.088
Benign	148 (67.0)	2 (25.0)	150 (65.5)	
Neuroendocrine tumor	1 (0.5)	0 (0)	1 (0.4)	
Poorly cohesive carcinoma	2 (0.9)	0 (0)	2 (0.9)	
Tubular adenocarcinoma	70 (31.7)	6 (75.0)	76 (33.2)	
Location, *n* (%)				0.262
Lower third	127 (57.5)	5 (62.5)	132 (57.6)	
Mid third	72 (32.6)	1 (12.5)	73 (31.9)	
Upper third	22 (10.0)	2 (25.0)	24 (10.5)	
Antiplatelet agents, *n* (%)	46 (20.8)	2 (25.0)	48 (21.0)	0.775
Anticoagulants, *n* (%)	9 (4.1)	0 (0)	9 (3.9)	0.560
NSAID use, *n* (%)	14 (6.3)	0 (0)	14 (6.1)	0.463
Diabetes mellitus, *n* (%)	113 (51.4)	6 (75.0)	119 (52.2)	0.189
Hypertension, *n* (%)	37 (16.8)	3 (37.5)	40 (17.5)	0.131

SD, standard deviation; ESD, endoscopic submucosal dissection; NSAID, nonsteroidal anti-inflammatory drug.

## 4. Discussion

In this retrospective single-center cohort of patients undergoing gastric ESD, we found that ilaprazole achieved artificial ulcer–healing outcomes comparable to those of esomeprazole at approximately 8-week follow-up. The overall complete healing rate exceeded 90% in both groups, and treatment allocation was not independently associated with incomplete healing. Instead, an artificial ulcer size ≥ 30 mm was the only independent predictor of delayed healing, whereas the treatment group was not associated with healing outcomes after adjustment. Post-discharge delayed bleeding occurred in 8 of 229 patients (3.5%), corresponding to 5.4% in the esomeprazole group and 0% in the ilaprazole group. Importantly, this incidence falls within the range of 3–11% reported in previous large ESD cohorts [3,6,9]. Although delayed bleeding was more frequently observed among patients with incomplete healing in univariate analysis, it did not retain statistical significance when included in the multivariate model evaluating predictors of ulcer healing, likely reflecting the limited number of bleeding events rather than a definitive causal relationship. Collectively, these findings suggest that lesion-related factors play a central role in post-ESD ulcer repair, while modern PPIs provide broadly comparable healing efficacy under routine clinical practice. An important aspect of our study design is that all patients received a uniform intravenous esomeprazole regimen during the immediate post-ESD period. Therefore, the comparison in this study reflects differences in oral maintenance therapy rather than the full peri-procedural acid suppression strategy. Given that the early post-procedural phase may be critical for initial hemostasis and mucosal stabilization, our findings should be interpreted within the context of a shared induction phase. Further studies comparing complete treatment regimens from the immediate postoperative period would be required to fully assess potential differences between PPIs.

The prominent role of artificial ulcer size in determining healing outcomes may be better understood by distinguishing ESD-induced ulcers from conventional peptic ulcers. In general clinical practice, most peptic ulcers are relatively smaller in surface extent but often involve deeper inflammatory injury extending into the submucosa and occasionally the proper muscle layer, accompanied by chronic inflammatory infiltration and fibrosis [4]. In contrast, artificial ulcers created during ESD are intentionally wide mucosal defects generated by controlled submucosal dissection. Although these ulcers are frequently larger in surface diameter than typical peptic ulcers, the surrounding tissue is usually free of longstanding inflammatory damage, and the muscularis propria is generally preserved unless procedural injury occurs. Histopathologic studies have demonstrated that artificial ulcers primarily undergo healing through early contraction of the resection base, followed by granulation tissue formation and re-epithelialization, a process that typically completes within 6–8 weeks under adequate acid suppression [4,7,10]. Therefore, in the absence of deep structural injury or severe submucosal fibrosis, most artificial ulcers are expected to heal within a predictable timeframe. However, when the resection area becomes extensive—such as in lesions ≥ 30 mm—the required degree of contraction and epithelial coverage increases substantially, which may explain why ulcer size emerged as the dominant determinant of delayed healing in our cohort. This finding aligns with prior studies identifying the resection area as a key predictor of artificial ulcer healing after gastric ESD [7,11].

Ilaprazole is characterized by sustained acid suppression and relatively low dependence on CYP2C19-mediated metabolism, resulting in more stable pharmacokinetic and pharmacodynamic profiles compared with several conventional PPIs [12,13]. These properties have raised the possibility that ilaprazole might confer advantages in promoting mucosal healing after gastric ESD. Although the CYP2C19 polymorphism is relatively common in East Asian populations, including Koreans [12], esomeprazole itself is less influenced by CYP2C19 variability than earlier-generation PPIs [14]. Therefore, the clinical impact of genotype-related metabolic differences may be attenuated in the comparison between ilaprazole and esomeprazole. In addition, once adequate acid suppression is achieved with standard-dose therapy, the incremental benefit of further pharmacologic potency may be limited in the context of artificial ulcers created by ESD. This may explain why ulcer-healing rates in our cohort were similarly high in both groups and why treatment allocation was not independently associated with healing outcomes. Our findings are consistent with prior studies demonstrating broadly comparable healing efficacy among contemporary PPIs when administered at appropriate doses and durations after ESD [6,15].

Post-discharge delayed bleeding remains one of the most clinically relevant adverse events following gastric ESD. In our cohort, delayed bleeding occurred in 8 of 229 patients (3.5%), corresponding to 5.4% in the esomeprazole group and 0% in the ilaprazole group. This incidence is consistent with previously reported bleeding rates of approximately 3–11% in large ESD series [3,6,9], suggesting that our cohort reflects real-world clinical practice rather than an outlier population. Although delayed bleeding was more frequently observed among patients with incomplete ulcer healing in univariate analysis, its independent association with healing could not be confirmed when incorporated into the multivariate model, likely due to the small number of events. Nevertheless, the relationship between delayed bleeding and subsequent ulcer healing warrants consideration from a mechanistic standpoint. All bleeding episodes in this study required endoscopic hemostasis using coagulation-based techniques. Thermal coagulation, while effective for immediate hemostasis, may induce additional tissue injury to the ulcer base, potentially extending local inflammation and delaying re-epithelialization. In artificial ulcers created by ESD—where healing relies heavily on coordinated contraction and epithelial regeneration—even limited additional injury could theoretically influence the repair process. Therefore, delayed bleeding may function not only as a clinical event but also as a marker of procedural complexity or secondary tissue insult. However, given the limited number of bleeding cases and the retrospective design, these observations should be interpreted cautiously and regarded as hypothesis-generating rather than definitive. In our cohort, post-discharge delayed bleeding was observed only in the esomeprazole group, whereas no events occurred in the ilaprazole group. However, this finding should be interpreted with caution, given the small number of events and the known multifactorial nature of post-ESD bleeding. Factors such as lesion size, procedural complexity, submucosal fibrosis, histology, and antithrombotic use may have contributed to the observed distribution of bleeding events. Therefore, this result should be considered exploratory and hypothesis-generating rather than indicative of a causal relationship between PPI type and bleeding risk. Further studies with larger sample sizes and adequately powered designs are required to clarify whether differences in acid-suppressive agents influence post-ESD bleeding risk.

Several limitations should be acknowledged. First, this was a retrospective single-center study, and treatment allocation was not randomized. Although baseline characteristics were largely comparable between groups, unmeasured confounders may have influenced both ulcer-healing outcomes and bleeding risk. Second, the number of incomplete healing cases and post-discharge delayed bleeding events was relatively small, which limited statistical power and may have contributed to wide confidence intervals in the multivariate analysis. In particular, only 13 patients experienced incomplete healing, and the inclusion of multiple variables in relation to this limited number of events may have resulted in model overfitting and unstable estimates. Therefore, the results of the multivariable analysis should be interpreted with caution and considered hypothesis-generating rather than definitive. In addition, the absence of bleeding events in the ilaprazole group precluded multivariate modeling for bleeding outcomes. Third, follow-up endoscopy was performed within a clinically acceptable window around 8 weeks rather than on a fixed date, reflecting real-world practice but introducing potential variability in healing assessment. Finally, intragastric pH monitoring or pharmacodynamic measurements were not performed; therefore, we cannot directly compare the acid-suppressive effects of the two PPIs in this cohort.

## 5. Conclusions

In conclusion, ilaprazole demonstrated ulcer-healing efficacy comparable to that of esomeprazole following gastric ESD, with high overall rates of complete artificial ulcer healing at approximately 8 weeks. Treatment allocation was not independently associated with healing outcomes, whereas artificial ulcer size ≥ 30 mm emerged as the principal determinant of delayed healing. The observed incidence of post-discharge delayed bleeding was consistent with previously reported ranges and, although associated with incomplete healing in univariate analysis, could not be confirmed as an independent predictor after adjustment. These findings suggest that lesion-related factors, rather than pharmacologic differences between modern PPIs, primarily influence artificial ulcer repair after gastric ESD. Ilaprazole may therefore be considered a reasonable maintenance PPI option in routine clinical practice, while careful attention should be given to resection extent and bleeding risk in post-ESD management.

## Data Availability

The data presented in this study are available on request from the corresponding author due to ethical reasons.

## Abbreviations

ESD, Endoscopic submucosal dissection; PPI, Proton pump inhibitor; OR, Odds ratio; CI, confidence interval.
